# Human Embryonic Stem Cell Differentiation Toward Regional Specific Neural Precursors

**DOI:** 10.1634/stemcells.2008-0543

**Published:** 2009-01

**Authors:** Slaven Erceg, Mohammad Ronaghi, Miodrag Stojković

**Affiliations:** Cellular Reprogramming Laboratory; Centro de Investigación Príncipe Felipe (CIPF)Valencia, Spain

**Keywords:** Human embryonic stem cells, Neural differentiation, Cell culture, Cellular therapy

## Abstract

Human embryonic stem cells (hESCs) are self-renewing pluripotent cells that have the capacity to differentiate into a wide variety of cell types. This potentiality represents a promising source to overcome many human diseases by providing an unlimited supply of all cell types, including cells with neural characteristics. Therefore, this review summarizes early neural development and the potential of hESCs to differentiate under in vitro conditions, examining at the same time the potential use of differentiated hESCs for therapeutic applications for neural tissue and cell regeneration.

## INTRODUCTION

Human embryonic stem cells (hESCs) have been successfully derived from early preimplantation human embryos [1] and have been shown to have a normal karyotype [2], express high levels of telomerase activity [3], and have specific pluripotent intracellular and cell surface markers, and can be propagated for extended periods of time [4]. They are self-renewing pluripotent cells that theoretically have the potential to differentiate into nearly all cell types of the human body [3, 4]. This potentiality represents a promising source to overcome many human diseases by providing an unlimited supply of all cell types, including neural cells and specific subtypes of neural precursors including mature oligodendrocytes, motoneurons, and dopaminergic (DA) cells for future cell-based therapies for neurodegenerative and neurological disorders.

**Figure 1:**
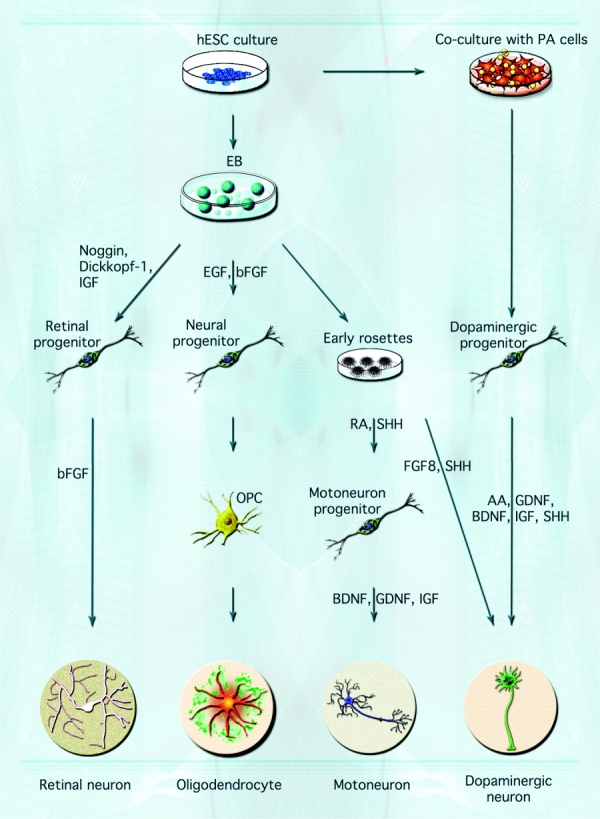
Steps of different protocols to induce neural differentiation of hESCs. hESCs can be differentiated into neuronal lineages using EBs. Prolonged treatment of EBs with EGF and bFGF in appropriate culture conditions results in the derivation of OPCs that can be used to repair injured spinal cord. Treatment of EBs with RA generates neuroectodermal cellular formations called rosettes. Cells from rosettes differentiate into motoneuron progenitors if triggered with RA and SHH, and can be a very useful tool in the future treatment of spinal cord injuries. Differentiation toward DA progenitors can be induced with FGF8 and SHH in the early rosette stage. Coculture of hESCs with mesenchymal PA6 cells results in a high yield of DA neurons. Abbreviations: AA, ascorbic acid; BDNF, brain-derived neurotrophic factor; bFGF, basic fibroblast growth factor; DA, dopaminergic; EB, embryoid body; EGF, epidermal growth factor; FGF, fibroblast growth factor; GDNF, glial-derived neurotrophic factor; hESC, human embryonic stem cell; IGF, insulin-like growth factor; OPC, oligodendrocyte progenitor cell; RA, retinoic acid; SHH, sonic hedgehog.

The neural differentiating pathway of hESCs can be induced and enhanced under in vitro conditions, and this can be achieved by adding growth factors, growth factor antagonists, and morphogens (Fig. 1) [5, 6]. However, the protocol, which includes selection, concentration, and the time point when an exogenous differentiation factor needs to be applied, is a very important issue in targeted differentiation of hESCs and should be considered precisely.

First, there have been studies that used spontaneous differentiation as a starting point to differentiate hESCs into highly purified neural lineages [5, 7]. The formation of ectodermal derivatives can be induced by prolonged culture of hESCs [5] without changing the feeder cells [4, 8]. With this strategy, the neural progenitors obtained could differentiate into the major central nervous system (CNS) lineages: oligodendrocytes, astrocytes, and neurons (Table 1). One of the most often used factors that promotes neuralization is retinoic acid (RA). However, the cell population obtained after application of this differentiation strategy is still relatively heterogeneous [5, 7]. The most well-studied hESC differentiation system involves the formation of three-dimensional structures called embryoid bodies (EBs) [9]. These structures appear when clumps of hESCs aggregate in culture dishes that do not favor cell adhesion or attachment. However, spontaneous differentiation of EBs yields only a small fraction of cells with neural lineages. Therefore, to induce neural differentiation EBs are treated with different morphogens and growth factors (Fig. 1). In addition, transfection, as a tool to express different transcription factors endogenously, and coculture of hESCs with different cell types that are capable of inducing a specific lineage or directing differentiation are additional strategies for targeted differentiation of hESCs.

**Table 1:** Human embryonic stem cell differentiation toward regional specific neural precursors using different protocols

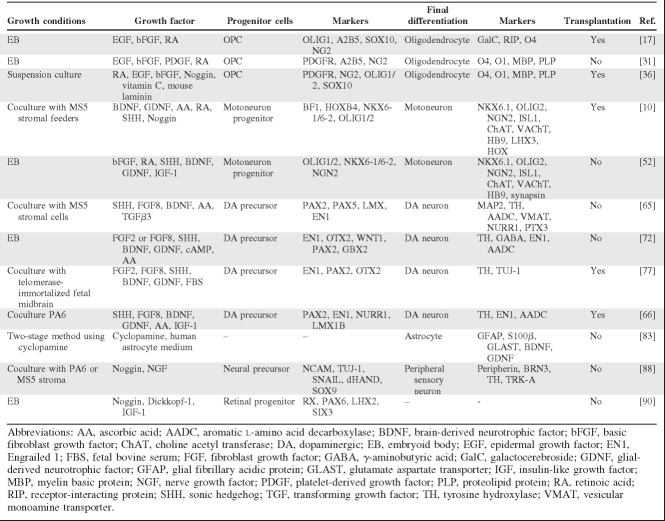

Abbreviations: AA, ascorbic acid; AADC, aromatic l-amino acid decarboxylase; BDNF, brain-derived neurotrophic factor; bFGF, basic fibroblast growth factor; ChAT, choline acetyl transferase; DA, dopaminergic; EB, embryoid body; EGF, epidermal growth factor; EN1, Engrailed 1; FBS, fetal bovine serum; FGF, fibroblast growth factor; GABA, γ-aminobutyric acid; GalC, galactocerebroside; GDNF, glial-derived neurotrophic factor; GFAP, glial fibrillary acidic protein; GLAST, glutamate aspartate transporter; IGF, insulin-like growth factor; MBP, myelin basic protein; NGF, nerve growth factor; PDGF, platelet-derived growth factor; PLP, proteolipid protein; RA, retinoic acid; RIP, receptor-interacting protein; SHH, sonic hedgehog; TGF, transforming growth factor; TH, tyrosine hydroxylase; VMAT, vesicular monoamine transporter.

Among the different coculture systems, stromal cells efficiently support the differentiation of hESCs mostly toward rostral neuronal progenitors [10], but further manipulation of these early progenitors by cell sorting and/or using different growth factors results in midbrain DA neurons [11], neural crest cells [12, 13], peripheral sensory neurons [13], and spinal motoneurons [10]. hESC-derived neurons in culture have been shown to respond to neurotransmitters and generate action potentials [5]. In addition, accumulating data [10, 14–17] have shown the therapeutic value of various neural precursor cells (NPCs) in experimental models of neurological diseases. The development of transplantable NPCs and cell lineages has had a great effect on biomedical research, already serving as a valuable system for developmental and translational research including drug and cell therapy development. Cell therapy for specific neural disorders or injuries requires the production of cells that are committed to specific neural lineages.

This review summarizes the potential of hESCs to differentiate under in vitro conditions and the potential use of differentiated neural cells for therapeutic applications for neural tissue and cell regeneration.

## DIFFERENTIATION OF hESCs TOWARD OLIGODENDROCYTES

Oligodendrocytes are non-neuronal cells located in the white matter and have a vital role in the support and maintenance of the CNS by insulating the axons of the nerve cells [18]. During the process of development, oligodendrocytes originate from the ectodermal germ layer and oligodendrocyte precursor cells (OPCs), which are induced from neuroepithelium [19]. These cells undergo proliferation, migration through the CNS, and finally differentiation toward mature oligodendrocytes. All these processes are exerted by the expression of specific transcription factors and local axonal signals [19].

Oligodendrocytes are very easily identifiable through a number of specific markers. The most important markers of OPCs and oligodendrocytes include NG2, a membrane chondroitin sulfate proteoglycan [20]; platelet-derived growth factor receptor α subunit (PDGFR-α) [21]; galactocerebroside (GalC), the marker for committed oligodendrocytes; myelin basic protein (MBP), the marker of mature myelin [22]; myelin proteolipid protein (PLP), the component of myelin that is expressed on oligodendrocytes and glial precursors [23]; O4, the marker for oligodendrocytes; and finally, oliogodendrocyte lineage genes (*OLIG*) [24]. Oligodendrocyte differentiation factors include ligands that bind the cell surface through nuclear thyroid hormone receptors. It seems that thyroid hormone can induce the expression of RA receptors too. Billon et al. [25] have shown that thyroid hormone receptor α-1 mediates normal differentiation and promotes the effect of this hormone on OPCs.

It has been shown that insulin, insulin-like growth factors (IGFs) [26], and epidermal growth factor (EGF) [27] play a crucial role in oligodendrocyte development and proliferation. Some studies in mice have shown that under in vitro conditions IGF-1 increases the number of mature oligodendrocytes as well as the proliferation of OPCs [28]. However, it seems that the most important factors that induce oligodendrocyte differentiation are ligands that bind to RA receptors and factors that activate the extracellular signal-regulated kinase pathway.

Most of these studies were performed in mice, and the lack of human data extrapolates these findings to hESCs. The only known human caudalizing growth factor is RA, exerting an opposing action to fibroblast growth factor (FGF) during rostrocaudal regional identity determination of spinal cord progenitor cells [29, 30]. Thus, oligodendrocyte differentiation from hESCs requires growth and transcription factors involved in the early phases of human neural development.

Differentiation of hESCs toward oligodendrocyte progenitors could be a good strategy for cell therapy in diseases affected by damage or disruption of the myelin sheath. Considerable effort has been put forth to derive oligodendrocytes from hESCs as a possible in vitro source for cell transplantation [17, 31, 32]. For the first time, Nistor et al. [32] reported a protocol using the EB step for the derivation of oligodendrocytes from hESCs. The authors observed that an 8-day exposure of EBs to RA resulted in the formation of three-dimensional structures, so called yellow spheres. These structures started to grow rapidly in glial restriction medium, which consists of different factors, including insulin, progesterone, selenium ions, thyroid hormone, and EGF. Differentiated OPCs expressed markers including OLIG1, SOX10, A2B5, and NG2, which demonstrated the oligodendroglial precursor lineage of the cells. After 42 days, the differentiated cells expressed GalC, NG2, and O4, which confirmed the presence of mature oligodendrocytes. The final population of differentiated OPCs revealed a small population of glial fibrillary acidic protein (GFAP)^+^ cells and neuron-specific βIII tubulin (TUJ1)^+^ cells, which suggests a pure population of OPCs with a very small portion of astrocytes and neurons. Another protocol for derivation of mature oligodendrocytes was developed by Kang et al. [31]. After the formation of EBs in the hESC medium without basic fibroblast growth factor (bFGF), the authors cultured EBs in the presence of *i*nsulin, *t*ransferrin, *s*elenium chloride, and *f*ibronectin (ITSF medium) [33]. The latter has been shown to have an essential role in cellular migration during neural crest development. The neural progenitors were selected after 5 days and cultured in a bFGF-containing medium to promote the proliferation and expansion of neural precursors. Rosette-like structures were mechanically isolated to form spherical neural masses (SNMs). These masses attached in Matrigel, and the presence of EGF and PDGF induced proliferation of neural precursors and early OPCs, respectively [34]. At this stage, the cells expressed oligodendrocyte precursor markers such as PDGFR, A2B5, and NG2. After removing the growth factors, thyroid hormone was added. The latter plays an important role during oligodendrocyte development via a mechanism that consists of two components: (a) a timing component, which depends on the mitogen (PDGF), and (b) an effector component, which depends on thyroid hormone and stops cell division and promotes oligodendrocyte differentiation at the appropriate time [35]. Upon the addition of thyroid hormone, mature oligodendrocytes differentiated from the precursors. Finally, all cells expressed oligodendrocyte-specific markers such as oligodendrocyte surface protein O4, O1, MBP, and PLP.

Differentiation of hESCs into mature oligodendrocytes can also be induced by the addition of the bone morphogenic protein (BMP) antagonist Noggin at a specific stage after the induction of neural precursor cells by RA [36]. The treatment of cells with Noggin stimulates myelin production of oligodendrocytes to a great extent. Unfortunately, this differentiation protocol is not cell-specific since differentiated neural cells were contaminated with cells that expressed specific endodermal markers. Under serum-free culture conditions, Noggin promotes the conversion of hESCs into astrocytes, oligodendrocytes, and electrophysiologically functional mature neurons, but during prolonged cell propagation the differentiation potential of the neural precursors shifts from a neuronal to a glial fate [37]. Therefore, to increase the efficiency of oligodendrocyte differentiation, hESCs have been grown in the presence of bFGF, FGF-4, EGF following exposure to PDGF, IGF-1, and factors that can elevate cAMP levels, such as forskolin [38]. In all these studies, extrinsic factors involved in dorsoventral patterning of the spinal cord were not used, leaving the possibility that these oligodendrocyte progenitors may not have specific spinal characteristics. The motoneuron domain (pMN) is a progenitor domain expressing the gene *OLIG2*, which has been shown to be involved in motoneuron and oligodendrocyte specification. Curiously, Kang et al. [31] and Nistor et al. [32] obtained highly pure oligodendrocyte populations without using these specific factors. Previous testing of these progenitors to *OLIG2*^+^ cells (the gene characteristic for the pMN domain) could be important in our understanding of whether an EB-based method could serve as a model to direct differentiation of rosettes to OPCs. Nevertheless, more studies have to be performed to confirm and reproduce these results.

Several studies have already demonstrated that transplantation of some oligodendroglial lineage cells resulted in the recovery of motor function and remyelination of the injury site [17, 32, 41, 42]. A very high purity OPC population was obtained after hESCs were treated with bFGF, RA, and EGF [32]. Animals grafted with OPCs exhibited enhanced remyelination and substantially improved locomotor ability 7 days after injury [17]. On the other hand, OPCs transplanted 10 months after spinal cord injury (SCI) survived and proliferated but they had neither the ability to remyelinate the axons nor the ability to improve locomotor ability [17]. Pathological analysis revealed the presence of astrogliosis, engulfment of axons by astrocytes and a higher density of demyelinated axons. Although the mentioned studies have proven that very pure populations of OPCs can be derived from hESCs, more studies have to be performed to ensure that the transplantation of hESC-derived OPCs can be an effective strategy in order to treat acute SCI patients in the near future.

## DIFFERENTIATION OF hESCs TOWARD SPINAL MOTONEURONS

**Figure 2:**
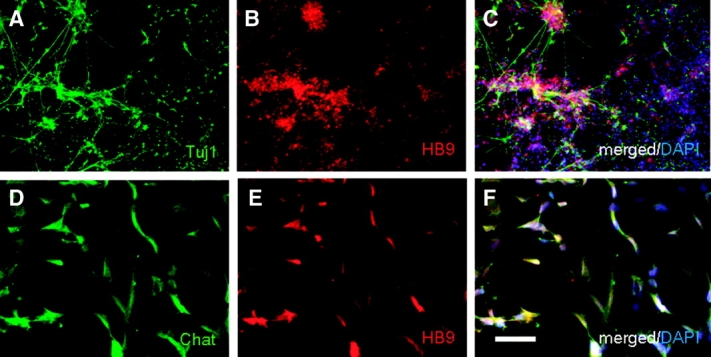
hESC-derived neural progenitors treated with retinoic acid display a spinal cord phenotype. The cells are mostly TUJ1^+^ (green, **(A)**) and HB9^+^ (red, **(B)**). Almost all ChAT^+^ cells (green, **(D)**) are also HB9^+^ cells (red, **(E)**). Blue indicates DAPI. Scale bars: 50 μm **(A–C)** and 25 μm **(D–F)**. Abbreviations: ChAT, choline acetyl transferase; DAPI, 4′,6-diamidino-2-phenylindole; hESC, human embryonic stem cell.

Neurons and glia are derived from the neuroectodermal part of the neural tube during early organogenesis. During development, some morphogens produce a positional code in a concentration gradient manner in different parts of the neural tube (dorsoventral or rostrocaudal) in order to force the cells to differentiate into different neural cells [43, 44]. It has been shown that spinal motoneurons are derived from a single pMN domain during development through the effect of sonic hedgehog (SHH) signaling pathways [45–47]. In the process of development these motoneurons can acquire different subtypes through a positional identification code in the spinal cord, which in turn is the result of the exposure of different concentrations of SHH and other morphogens and growth factors. Based on these facts, several protocols have been developed combining different morphogens and growth factors in different concentrations in order to obtain spinal cord motoneurons from hESCs [48, 49]. One study [50] confirmed that RA signaling causes a very strong level of caudalization in the spinal cord and induces differentiation of caudal CNS specification. This is very important since motoneurons are derived from the caudal and ventral parts of the neural tube, where RA induces motoneuron differentiation via genes such as *NEUROM* [51]. The role of RA in the differentiation of hESCs toward motoneurons has been confirmed [52], where chemically defined conditions resulted in the derivation of electrophysiologically active motoneurons that expressed HB9, HOXC8, and choline acetyl transferase (ChAT) (Fig. 2). Briefly, after the formation of EBs and changing the culture plate to a normal adherent plate in the presence of a neural induction medium containing bFGF, F12/Dulbecco's modified Eagle's medium, N2 supplement (recombinant insulin, human transferrin, sodium selenite, putrescine, and progesterone), and heparin, the cells showed specific columnar structures. After several days in the presence of RA, the attached neuroectodermal rosette-like structures were isolated and then successively treated with RA and SHH. The addition of brain-derived neurotrophic factor (BDNF), glial-derived neurotrophic factor (GDNF), and IGF-1 to the medium during 3 weeks in laminin/ornithine plates converted progenitors into mature neurons. These factors have previously been shown to enhance motoneuron differentiation under in vitro conditions [53, 54]. In the mentioned study [52], early rosettes expressed PAX6 but not SOX1, although after an additional 2 weeks in culture neural tube-like rosettes expressed both PAX6 and SOX1 [55]. Through the addition of RA and then SHH, after a period of 4 weeks, a large population of the cells expressed HB9, which has been shown to be a specific motor neuron transcription factor [56]. Only exposure of early-stage rosettes to these factors resulted in increased specific motoneuron differentiation. Coexpression of HB9 with Islet1 and LIM3, transcription factors related to the specific motoneuron genotype [57, 58], confirmed the motoneuron character of these cells. Whereas, in a concentration-dependent manner, RA induces the rostrocaudal characterization of neural tube cells, SHH [46, 59] and BMPs help neural tube cells to specify dorsoventrally. A very important role of the morphogenic factors RA and SHH in neural development was confirmed [10, 49], where both factors induced caudalization and promoted ventralization of hESCs. In a recently published report [60], it was demonstrated that a small molecule, purmorphamine, which activates the SHH pathway, induces directed differentiation of ventral spinal progenitors and motor neurons from hESCs. A genomewide gene expression analysis revealed that in vitro differentiated hESCs show a multifold increase in the expression of some motoneuron specification markers, including *HLXB9, NKX6-1, LHX3, OLIG2*, and *NKX2.2* and also HOX and RA-related genes [15, 49], *PAX6, NKX6-1/6-2, OLIG1/2*, and *NGN2* [56].

Unfortunately, these protocols did not result in pure populations of motoneuron precursors, inevitably increasing the risks associated with transplantation of undifferentiated and potentially neoplastic cells. A good strategy for obtaining pure populations of motoneuron progenitors could be transfection of hESCs. Nearly pure populations of motoneuron precursors have been obtained from differentiating and purifying hESCs previously transfected with plasmid carrying the green fluorescent protein gene (*GFP*) under the control of an enhancer element associated with the HB9 promoter [61]. Almost 90% of the cells were immunopositive for HB9, Islet1, and ChAT, and showed electrophysiological activity specific to motoneuron progenitors. Final differentiation was performed by plating GFP^+^ cells on freshly isolated skeletal muscle, where they formed functional neuromuscular connections, demonstrating the potential of differentiated hESCs for future stem cell-based therapies of SCI or other neurodegenerative disorders related to loss of motoneurons.

## DIFFERENTIATION OF hESCs TOWARD DA NEURONS

One of the most prominent human neurological disorders is Parkinson's disease, which is characterized by progressive and selective loss of DA neurons, caused by the insufficient formation and action of dopamine, which is produced in the DA neurons in midbrain substantia nigra [62]. DA neurons play a crucial role in the control of many brain functions, such as voluntary movements and many behavioral processes [63]. These neurons can be identified via the expression of some specific transcription factors, including Engrailed 1 (EN1), PITX3, NURR1, and LMX1b, which are also very important in the development of DA neurons [64].

**Figure 3:**
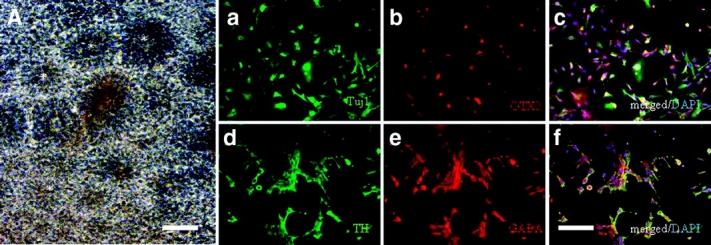
Differentiation of hESCs into DA neurons. **(A):** Early rosettes (marked by asterisks, bright light) and specific staining of regional specific neural precursors generated from hESCs in chemically defined medium conditions. The hESC-derived neural progenitors display a rostral phenotype if they are treated with bFGF only. The cells are TUJ1^+^ (green, **a**) coexpressed with OTX2^+^ (red, **b**), and a majority of the TH^+^ cells (green, **d)** are GABA^+^ (red, **e**). Scale bars: 25 μm **(A)** and 50 μm **(a–f)**. Abbreviations: bFGF, basic fibroblast growth factor; DA, dopaminergic; GABA, γ-aminobutyric acid; hESC, human embryonic stem cell; TH, tyrosine hydroxylase.

The limited availability of human cells and complicating dyskinesias after fetal or adult stem cell transplantation could be major limitations of this mode of therapy approach. hESCs, with their capacity for unlimited expansion and multilineage differentiation under in vitro growth conditions, could solve these problems. One important strategy to enhance the differentiation of hESCs toward the DA neuron lineage is coculture of hESCs with mouse bone marrow mesenchymal PA6 or MS5 stromal cells (Table 1) [11, 65, 66]. This effect of PA6 cells has been named the inductive factor stromal cell-derived inducing activity (SDIA) [67]. It seems that PA6 cells do not have the general capacity to promote specific differentiation into DA neurons from all types of neural stem cells in the same way as from hESCs. Some transcription factors, including NURR1, LMX1b, and PITX3, are essential for the development of midbrain DA neurons [68–70], including SHH and FGF8, if applied at very early stages of neuralization [71]. In addition, the percentage of typical neurons that express the enzyme tyrosine hydroxylase (TH) (Fig. 3) highly depends on the exposure to FGF and SHH during the 2 weeks after the beginning of neuralization [71].

Differentiation of hESCs toward DA neurons is usually performed via the formation of EBs [72]. After transfer of EBs from a low attachment plate into a normal adhesion plate, the EBs form neuroepithelial cells that organize into neural tube-like rosettes. After dissociation of neuroepithelial cells and addition of neural differentiation medium, which consists of BDNF, GDNF, AMP, and ascorbic acid (AA), DA differentiation begins 3–4 weeks after the initial treatment of hESCs [72]. The early rosettes differentiate toward late neural tube-like rosettes in the presence of bFGF or FGF8, and after 6 days of exposure to both factors the withdrawal of all morphogens results in the derivation of DA precursors. Cells treated with FGF2 in the early rosette stage form forebrain DA neurons whereas cells treated with FGF8 differentiate toward midbrain DA neurons. The latter treatment results in expression of *EN1, OTX2, WNT1, PAX2*, and *GBX2*, which are essential in the patterning of mid–hindbrain junctions [73, 74]. Additionally, the coexpression of TH and EN1 markers in early FGF8 treatment cultures has been observed, but not in early bFGF treatment cultures [73]. In contrast, the treatment of early rosettes with bFGF then with FGF8 and SHH leads to the differentiation of the cells into forebrain DA neurons, which are able to coexpress γ-aminobutyric acid (GABA) and TH but not EN1, confirming the forebrain character of these cells [72].

An additional method to obtain midbrain DA and TH^+^ neurons is the growth of EBs in a conditioned medium with a human hepatocarcinoma cell line followed by conventional serum-free culture in a medium containing bFGF [75, 76], or by coculturing them with telomerase-immortalized fetal midbrain astrocytes (Table 1) [77]. EBs plated on tissue culture dishes and in the presence of serum-free ITSF medium show induced differentiation toward DA precursors within 10 days. In the next step, the cells were transferred to polyornithine/laminin-coated dishes and exposed to a new medium supplemented with FGF2 and SHH [77]. Withdrawal of these factors, but growth of cells in the presence of BDNF, GDNF, and fetal bovine serum, yields DA neurons that are TH^+^. Most TH^+^ cells coexpress G-protein-gated inwardly rectifying K^+^ channel type 2 [78], which is almost exclusively expressed in the membrane of DA neurons projecting to the dorsolateral putamen, and are functionally linked to dopamine D2 and GABA_B_ receptors [78].

Although the intrinsic control of DA fate specification remains to be clarified, these results suggest that the FGF and SHH pathways play an instructive role and promote the differentiation of DA neurons from hESC-derived neuroepithelia (Fig. 1). This was confirmed in a recent report [79], where EB-based conditions were used in order to differentiate hESCs into TH^+^ cells. EBs were transferred to Matrigel-coated dishes for 5 days in order to produce NPCs. After 4 days and exposure to bFGF and N2, NPCs expressed Musashi and nestin and formed rosettes. Neural rosettes were mechanically isolated and cultured for an additional 10 days in low attachment plates in the presence of bFGF. The SNMs formed were maintained for several weeks with the capability of differentiation into DA neurons [79]. By transferring SNMs to Matrigel-coated plates, the cells displayed neuronal morphologies and expressed mature neuronal markers including βIII tubulin and NeuN. For DA neuron differentiation induction, the cells were treated for 4 days with SHH and FGF8, then the cells continued differentiation in the presence of AA for the next 6 days. More than 85% of the differentiated cells were DA neurons. A protocol that is based on a protocol of Sonntag et al. [78] showed that it is possible to enhance the development of neuroectodermal precursors and further the amount midbrain DA neurons by adding Noggin to the medium for the first 7 or 21 days of differentiation and during stromal feeder cell-based neuroectodermal induction [78].

It is easy to conclude that nearly all differentiation protocols are very similar but also differ in duration and/or the differentiation condition used (defined versus nondefined). Therefore, it is not surprising that the reports of cell engraftment after transplantation are controversial too. Some studies clearly demonstrated that very few DA neurons survive when transplanted in the stratum of hemi-Parkinsonian rats and therefore failed to improve behavioral deficits in animal models [66]. On the other hand, decent efficiency of DA neurons grafted in the stratum of Parkinsonian rats was demonstrated when generated NPCs obtained from spontaneously differentiated hESCs survived for at least 12 weeks after cell transplantation [16]. In addition, the grafted NPCs differentiated in vivo into DA neurons, resulting in a significant partial behavioral improvement in treated animals [16], which is similar to when transplanted DA neurons caused a significant, substantial, and long-lasting improvement in rat motor function [77]. Transplantation of hESCs previously cocultured with PA6 cells [80] or treated with Noggin [78] into the striatum of 6-hydroxydopamine treated Parkinsonian rats resulted in engraftment of differentiated DA neurons expressing specific neural markers [80]. The latter study clearly demonstrated that differentiated NPCs behave differently when implanted at different time points. Animals treated with 16-day-old NPCs formed teratomas, but the rats treated with the other two groups of NPCs (20 and 23 days in culture) remained healthy [80], which means that prolonged in vitro differentiation of hESCs is crucial for tumor prevention. Because of the very important roles of DA neurons in motor function modulation and its degeneration in Parkinson's disease, these cells are therefore one of the most interesting neural lineages for possible transplantation and cell replacement in human therapy. Although some protocols have been developed in order to differentiate hESCs into DA neurons, efficient pure generation of these cells has not yet been achieved. It is obvious that there is a need for many more studies to overcome these problems during in vitro differentiation and before cell transplantation.

## DIFFERENTIATION OF hESCs TOWARD OTHER NEURAL CELL TYPES

### Astrocytes

One of the most important cell types of the CNS is the astrocyte, which has a crucial supportive function during its development, secreting different neurotrophic factors including GDNF and BDNF [81, 82]. Astrocytes express specific astroglial markers, including GFAP and S100β, and targeted differentiation of hESCs into astrocytes has been described where a two-stage growth method without the formation of EBs was applied [83]. When undifferentiated hESCs were treated with cyclopamine for 4 days, differentiated cells showed typical decreased expression of MAP2 and TUJ1, but radial glial cells and astrocyte markers, including glutamate aspartate transporter, were substantially increased. This application of cyclopamine, which is a known SHH inhibitor, resulted in the generation of nearly 70% nestin-expressing and 78% GFAP-expressing cells.

### Peripheral Neurons

The necessity to produce peripheral neurons from hESCs is obvious since these cells are a promising source for the treatment of some peripheral neuropathies such as familial dysautonomia, a disease caused by mutations in the *IKBKAP* gene, which leads to degeneration of peripheral sensory neurons (PSNs) [84]. The protocol to differentiate hESCs to PSNs, sympathetic neurons, and neural crest cells was previously described applying an “SDIA-based” method and coculturing hESCs with mouse stromal PA6 cells [12]. After 7 days, the differentiated cells expressed NCAM, an NPC marker, and after 2 weeks many cells were TUJ1^+^ coexpressing peripherin, characteristic of neurons with peripheral axons [85]. In addition, the presence of BRN3, characteristic of PSNs [86], and the coexpression of peripherin and TH demonstrated the presence of sympathetic neurons [12, 87]. A recent study [88] demonstrated that the yield of PSNs can be efficiently increased by coculturing hESC-derived NPCs with PA6 stromal cells and in the presence of Noggin. The most recent study of Lee et al. [13] describes the conditions to direct hESCs into neurons of neural crest identity and their further conversion to peripheral neurons. Coculture with stromal MS5 cells spontaneously converts hESCs to rosettes. The authors showed that neural crest precursors spontaneously emerge in cultures of hESC-derived neural rosettes, and that their number can be regulated by adding extrinsic signals, such as FGF2 and BMP2, involved in the specification of neural crest development. After cell sorting using the specific markers for neural crest cells (p75/HNK1), sorted cells showed typical neural crest identity, expressing BRN3a, AP2, *PAX3*, and SNAIL. To expand the neural crest cells, they were cultured in the presence of FGF2 and EGF. To assess the differentiation potential of hESC-derived neural crest progenitors, neuronal differentiation was continued by withdrawal of FGF2/EGF and exposure to BDNF, GDNF, nerve growth factor, and dibutyryl cAMP, yielding peripheral sympathetic neurons (TH^+^/peripherin^+^) and sensory neurons (BRN3a^+^/peripherin^+^). This study showed that the neural fate of the cells could already be determined in the early phases of in vitro differentiation at the rosette stage. The findings that hESC-derived rosettes can be isolated, regionally specified, and expanded were confirmed by the same group [89]. It seems that future studies have to be focused on molecular mechanisms that control formation of rosettes, since these cells have different capacities to generate different types of neural cells by cell sorting and several growth factors and morphogens [10, 13, 89]. Nevertheless, further studies have to define other methods to derive rosettes, avoiding coculture with stromal cells. Developing the protocols that use animal-free ingredients and feeder-free conditions is for sure a more convenient way.

### Retinal Progenitor Cells

**Figure 4:**
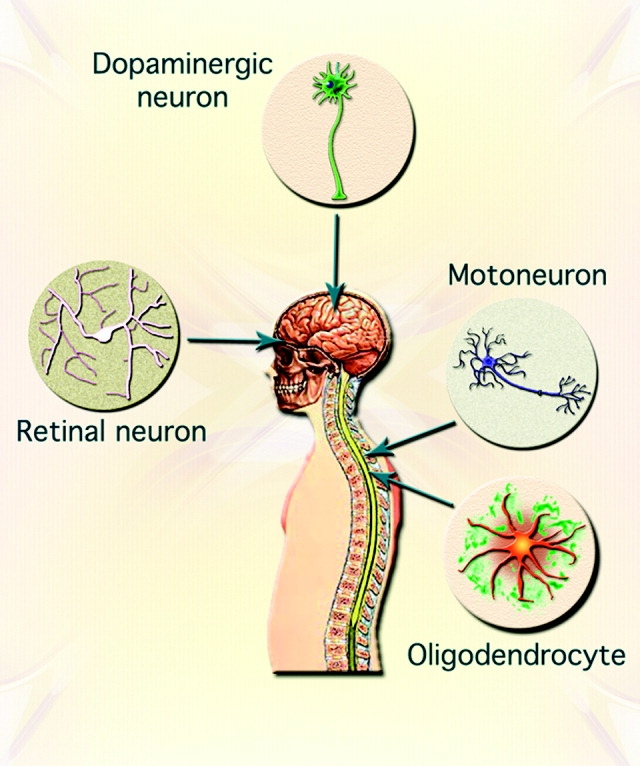
Potential cell therapy with differentiated human embryonic stem cells: retinal neurons for retinitis pigmentosa, dopaminergic neurons for Parkinson's disease, and motoneurons and oligodendrocytes for spinal cord injury.

hESCs have the ability to efficiently differentiate (approximately 80%) into retinal progenitor cells [90], and therefore could be used for the treatment of retinitis pigmentosa (Fig. 4), a disease caused by the degeneration of the neural retina [91]. In one study, EBs were treated for 3 days with a combination of factors including Noggin, Dickkopf-1 (Dkk1), and IGF-1 (Fig. 1), which can bind and inactivate members of the TGFβ superfamily of signaling proteins, and the Wnt signaling pathway [92]. Furthermore, the cells were transferred to Matrigel-coated plates in the presence of bFGF. After 3 weeks, the produced cells expressed eye field transcription factors such as RX, PAX6, LHX2, and SIX3, which showed the retinal progenitor identity of the differentiated cells. A recent study [93] used the EB-based protocol using the Wnt antagonist Dkk1 and Nodal antagonist Lefty-A during the first 20 days of the experimental procedure to obtain retinal pigment epithelial (RPE) cells from hESCs. After a 20-day treatment, the cells were plated on polyornithine/laminin matrix and maintained for 1 month. Typical morphological characteristics of mature RPE cells were observed after 50 days [93]. Also, additional treatment on day 25 with RA and taurine converted Dkk1/Lefty-A-treated EBs into photoreceptor precursors (CRX^+^ cells). Prolonged maintenance of cell culture (up to 200 days) increased the yield of these cells. Although these studies have shown that it is possible to generate an efficient protocol to obtain photoreceptors from hESCs, there are obstacles in obtaining very pure and a sufficient quantity of photoreceptor progenitors for future medical treatment.

## CONCLUSION

The challenge in using hESCs for developmental biology research and their possible application in regenerative medicine is to direct their wide differentiation potential into specific neural cell lineages. The most important concern of the recently published protocols of hESC differentiation toward specific neural lineages is the risk of contamination with non-neural cells, which limits the specificity of the differentiation protocol, efficiency, and eventual medical application of differentiated hESCs. As mentioned before, the use of stromal cell lines (PA6 or MS5), Matrigel, or conditioned medium, including a multistep procedure that involves the formation of EBs, bears the risk of pathogen cross-transfer or contamination with non-neural cells. In all protocols, the presence of mesodermal- and endodermal-originated cell lineages is inevitable, which is undesirable for further application in regenerative medicine. Therefore, considerable efforts need to be concentrated to develop defined and feeder-free conditions for differentiation of hESCs toward neural lineages.

In our recent work [30], we described growth conditions for the efficient and directed differentiation of hESCs toward very defined neural lineages. This in vitro system includes the use of feeder-free conditions, chemically defined medium, and the growth of differentiated hESCs without the formation of EBs. Our protocol involves the formation of rosettes (Fig. 3) and neural tube-like structures that can efficiently differentiate into neurons and glia. The rosette-derived progenitors formed bipolar NPCs that were positive for TUJ1, Musashi, nestin, A2B5, and MAP2. These progenitors were able to give rise to all three major neural lineages: neurons, astrocytes, and oligodendrocytes. The yield of neural progenitors was the same or even higher than in previously published protocols where chemically defined media and adherent conditions were used [94, 95], reaching >90% of the total cells after 8 weeks of differentiation [30]. The neurons obtained in this protocol revealed a more rostral character, expressing OTX2 (Fig. 3). Immunocytochemical analysis showed further evidence of the DA phenotype of these neurons with forebrain characteristics, because the majority of the neurons coexpressed TH and GABA (Fig. 3). By exposing the progenitor cells to RA at an early stage of the differentiation protocol, neural differentiation to the rostral forebrain dopamine neural lineage was suppressed and that of spinal neural tissue, including functional motor neurons, was promoted. In that study, we demonstrated that the use of extracellular inductive signals, more specifically RA, permits the efficient differentiation of hESCs into specific classes of CNS neurons. The only component of animal origin used in this protocol is B27 supplement, but it was shown that the presence of immunogenic nonhuman sialic acid is very low in hESC-derived neural precursors differentiated with this supplement [96]. It seems that the early phases of human neural development are the key to understanding the differentiation mechanisms toward more specific neural lineages. The focus of future investigations will be to understand the organization and regional specification of the first neuroepithelial structures, rosettes, that appear in the first phase of in vitro neural differentiation. This specific cellular arrangement, which resembles a sagittal view of the neural tube closely mimicking the neural tube stage, could bring us information about the differentiation fate of each rosette cell. As we can conclude from various neural differentiation protocols, the majority of these protocols include rosettes as a starting point. It will be critical to test the cellular organization of the rosette structure as well as the molecular specification of each rosette's cellular population to predict their differentiation fate, although it is still unclear whether these cells are capable of giving rise to the full cellular diversity of the human nervous system. In parallel, future studies have to focus on more simple and directed animal-free protocols to generate rosette cells. Additionally, there is a lack of efficient and robust protocols to obtain pure cell populations without the presence of other cell types, or worse, with the presence of undifferentiated hESCs. Therefore, the development of reliable and reproducible protocols for targeted differentiation of hESCs toward cells with neural characteristics will result not only in improved cell therapy but also in more efficient drug development, toxicology screening, and basic developmental studies.

## References

[1] Thomson JA, Itskovitz-Eldor J, Shapiro SS (1998). Embryonic stem cell lines derived from human blastocysts. Science.

[2] Buzzard JJ, Gough NM, Crook JM (2004). Karyotype of human ES cells during extended culture. Nat Biotechnol.

[3] Heins N, Englund MC, Sjöblom C (2004). Derivation, characterization, and differentiation of human embryonic stem cells. Stem Cells.

[4] Reubinoff BE, Pera MF, Fong CY (2000). Embryonic stem cell lines from human blastocysts: Somatic differentiation in vitro. Nat Biotechnol.

[5] Carpenter MK, Inokuma MS, Denham J (2001). Enrichment of neurons and neural precursors from human embryonic stem cells. Exp Neurol.

[6] Trounson A (2006). The production and directed differentiation of human embryonic stem cells. Endocr Rev.

[7] Schuldiner M, Yanuka O, Itskovitz-Eldor J (2000). Effects of eight growth factors on the differentiation of cells derived from human embryonic stem cells. Proc Natl Acad Sci U S A.

[8] Reubinoff BE, Itsykson P, Turetsky T (2001). Neural progenitors from human embryonic stem cells. Nat Biotechnol.

[9] Itskovitz-Eldor J, Schuldiner M, Karsenti D (2000). Differentiation of human embryonic stem cells into embryoid bodies compromising the three embryonic germ layers. Mol Med.

[10] Lee H, Shamy GA, Elkabetz Y (2007). Directed differentiation and transplantation of human embryonic stem cell-derived motoneurons. Stem Cells.

[11] Vazin T, Chen J, Lee CT (2008). Assessment of stromal-derived inducing activity in the generation of dopaminergic neurons from human embryonic stem cells. Stem Cells.

[12] Pomp O, Brokhman I, Ben-Dor I (2005). Generation of peripheral sensory and sympathetic neurons and neural crest cells from human embryonic stem cells. Stem Cells.

[13] Lee G, Kim H, Elkabetz Y (2007). Isolation and directed differentiation of neural crest stem cells derived from human embryonic stem cells. Nat Biotechnol.

[14] Zhang SC, Wernig M, Duncan ID (2001). In vitro differentiation of transplantable neural precursors from human embryonic stem cells. Nat Biotechnol.

[15] Ben-Hur T (2006). Human embryonic stem cells for neuronal repair. Isr Med Assoc J.

[16] Ben-Hur T, Idelson M, Khaner H (2004). Transplantation of human embryonic stem cell-derived neural progenitors improves behavioral deficit in Parkinsonian rats. Stem Cells.

[17] Keirstead HS, Nistor G, Bernal G (2005). Human embryonic stem cell-derived oligodendrocyte progenitor cell transplants remyelinate and restore locomotion after spinal cord injury. J Neurosci.

[18] Baumann N, Pham-Dinh D (2001). Biology of oligodendrocyte and myelin in the mammalian central nervous system. Physiol Rev.

[19] Miller RH (2002). Regulation of oligodendrocyte development in the vertebrate CNS. Prog Neurobiol.

[20] Polito A, Reynolds R (2005). NG2-expressing cells as oligodendrocyte progenitors in the normal and demyelinated adult central nervous system. J Anat.

[21] McKinnon RD, Waldron S, Kiel ME (2005). PDGF alpha-receptor signal strength controls an RTK rheostat that integrates phosphoinositol 3′-kinase and phospholipase Cgamma pathways during oligodendrocyte maturation. J Neurosci.

[22] Shiota C, Miura M, Mikoshiba K (1989). Developmental profile and differential localization of mRNAs of myelin proteins (MBP and PLP) in oligodendrocytes in the brain and in culture. Brain Res Dev Brain Res.

[23] Nadon NL, West M (1998). Myelin proteolipid proteFunction in myelin structure is distinct from its role in oligodendrocyte development. Dev Neurosci.

[24] Lu QR, Park JK, Noll E (2001). Oligodendrocyte lineage genes (OLIG) as molecular markers for human glial brain tumors. Proc Natl Acad Sci U S A.

[25] Billon N, Jolicoeur C, Tokumoto Y (2002). Normal timing of oligodendrocyte development depends on thyroid hormone receptor alpha 1 (TRalpha1). EMBO J.

[26] Barres BA, Hart IK, Coles HS (1992). Cell death and control of cell survival in the oligodendrocyte lineage. Cell.

[27] Ivkovic S, Canoll P, Goldman JE (2008). Constitutive EGFR signaling in oligodendrocyte progenitors leads to diffuse hyperplasia in postnatal white matter. J Neurosci.

[28] McMorris FA, Dubois-Dalcq M (1988). Insulin-like growth factor I promotes cell proliferation and oligodendroglial commitment in rat glial progenitor cells developing in vitro. J Neurosci Res.

[29] Bel-Vialar S, Itasaki N, Krumlauf R (2002). Initiating Hox gene expression: In the early chick neural tube differential sensitivity to FGF and RA signaling subdivides the HoxB genes in two distinct groups. Development.

[30] Erceg S, Lainez S, Ronaghi M (2008). Differentiation of human embryonic stem cells to regional specific neural precursors in chemically defined medium conditions. PloS ONE.

[31] Kang SM, Cho MS, Seo H (2007). Efficient induction of oligodendrocytes from human embryonic stem cells. Stem Cells.

[32] Nistor GI, Totoiu MO, Haque N (2005). Human embryonic stem cells differentiate into oligodendrocytes in high purity and myelinate after spinal cord transplantation. Glia.

[33] Okabe S, Forsberg-Nilsson K, Spiro AC (1996). Development of neuronal precursor cells and functional postmitotic neurons from embryonic stem cells in vitro. Mech Dev.

[34] Baron W, Metz B, Bansal R (2000). PDGF and FGF-2 signaling in oligodendrocyte progenitor cells: Regulation of proliferation and differentiation by multiple intracellular signaling pathways. Mol Cell Neurosci.

[35] Durand B, Raff M (2000). A cell-intrinsic timer that operates during oligodendrocyte development. Bioessays.

[36] Izrael M, Zhang P, Kaufman R (2007). Human oligodendrocytes derived from embryonic stem cells: Effect of noggin on phenotypic differentiation in vitro and on myelination in vivo. Mol Cell Neurosci.

[37] Itsykson P, Ilouz N, Turetsky T (2005). Derivation of neural precursors from human embryonic stem cells in the presence of noggin. Mol Cell Neurosci.

[38] Pic P, Michel-Bechet M, el Atiq F (1986). Forskolin stimulates cAMP production and the onset of the functional differentiation in the fetal rat thyroid in vitro. Biol Cell.

[39] Kakulas BA (1999). The applied neuropathology of human spinal cord injury. Spinal Cord.

[40] Totoiu MO, Keirstead HS (2005). Spinal cord injury is accompanied by chronic progressive demyelination. J Comp Neurol.

[41] Utzschneider DA, Archer DR, Kocsis JD (1994). Transplantation of glial cells enhances action potential conduction of amyelinated spinal cord axons in the myelin-deficient rat. Proc Natl Acad Sci U S A.

[42] Warrington AE, Barbarese E, Pfeiffer SE (1993). Differential myelinogenic capacity of specific developmental stages of the oligodendrocyte lineage upon transplantation into hypomyelinating hosts. J Neurosci Res.

[43] Jessell TM (2000). Neuronal specification in the spinal cord: Inductive signals and transcriptional codes. Nat Rev Genet.

[44] Briscoe J, Ericson J (2001). Specification of neuronal fates in the ventral neural tube. Curr Opin Neurobiol.

[45] Danesin C, Agius E, Escalas N (2006). Ventral neural progenitors switch toward an oligodendroglial fate in response to increased Sonic hedgehog (Shh) activity: Involvement of Sulfatase 1 in modulating Shh signaling in the ventral spinal cord. J Neurosci.

[46] Briscoe J, Chen Y, Jessell TM (2001). A hedgehog-insensitive form of patched provides evidence for direct long-range morphogen activity of sonic hedgehog in the neural tube. Mol Cell.

[47] Ericson J, Briscoe J, Rashbass P (1997). Graded sonic hedgehog signaling and the specification of cell fate in the ventral neural tube. Cold Spring Harb Symp Quant Biol.

[48] Christou YA, Moore HD, Shaw PJ (2007). Embryonic stem cells and prospects for their use in regenerative medicine approaches to motor neurone disease. Neuropathol Appl Neurobiol.

[49] Shin S, Dalton S, Stice SL (2005). Human motor neuron differentiation from human embryonic stem cells. Stem Cells Dev.

[50] Irioka T, Watanabe K, Mizusawa H (2005). Distinct effects of caudalizing factors on regional specification of embryonic stem cell-derived neural precursors. Brain Res Dev Brain Res.

[51] Maden M (2006). Retinoids and spinal cord development. J Neurobiol.

[52] Li XJ, Du ZW, Zarnowska ED (2005). Specification of motoneurons from human embryonic stem cells. Nat Biotechnol.

[53] Zurn AD, Winkel L, Menoud A (1996). Combined effects of GDNF, BDNF, and CNTF on motoneuron differentiation in vitro. J Neurosci Res.

[54] Bréjot T, Blanchard S, Hocquemiller M (2006). Forced expression of the motor neuron determinant HB9 in neural stem cells affects neurogenesis. Exp Neurol.

[55] Wilson PG, Stice SS (2006). Development and differentiation of neural rosettes derived from human embryonic stem cells. Stem Cell Rev.

[56] Arber S, Han B, Mendelsohn M (1999). Requirement for the homeobox gene Hb9 in the consolidation of motor neuron identity. Neuron.

[57] Hutchinson SA, Eisen JS (2006). Islet1 and Islet2 have equivalent abilities to promote motoneuron formation and to specify motoneuron subtype identity. Development.

[58] Thaler JP, Lee SK, Jurata LW (2002). LIM factor Lhx3 contributes to the specification of motor neuron and interneuron identity through cell-type-specific protein-protein interactions. Cell.

[59] Soundararajan P, Lindsey BW, Leopold C (2007). Easy and rapid differentiation of embryonic stem cells into functional motoneurons using sonic hedgehog-producing cells. Stem Cells.

[60] Li XJ, Hu BY, Jones SA (2008). Directed differentiation of ventral spinal progenitors and motor neurons from human embryonic stem cells by small molecules. Stem Cells.

[61] Singh Roy N, Nakano T, Xuing L (2005). Enhancer-specified GFP-based FACS purification of human spinal motor neurons from embryonic stem cells. Exp Neurol.

[62] Chinta SJ, Andersen JK (2005). Dopaminergic neurons. Int J Biochem Cell Biol.

[63] Maxwell SL, Li M (2005). Midbrain dopaminergic development in vivo and in vitro from embryonic stem cells. J Anat.

[64] Smidt MP, Smits SM, Burbach JP (2003). Molecular mechanisms underlying midbrain dopamine neuron development and function. Eur J Pharmacol.

[65] Perrier AL, Tabar V, Barberi T (2004). Derivation of midbrain dopamine neurons from human embryonic stem cells. Proc Natl Acad Sci U S A.

[66] Park CH, Minn YK, Lee JY (2005). In vitro and in vivo analyses of human embryonic stem cell-derived dopamine neurons. J Neurochem.

[67] Mizuseki K, Sakamoto T, Watanabe K (2003). Generation of neural crest-derived peripheral neurons and floor plate cells from mouse and primate embryonic stem cells. Proc Natl Acad Sci U S A.

[68] Simon HH, Bhatt L, Gherbassi D (2003). Midbrain dopaminergic neurons: Determination of their developmental fate by transcription factors. Ann N Y Acad Sci.

[69] Smits SM, Smidt MP (2006). The role of Pitx3 in survival of midbrain dopaminergic neurons. J Neural Transm Suppl.

[70] Zeng X, Cai J, Chen J (2004). Dopaminergic differentiation of human embryonic stem cells. Stem Cells.

[71] Ye W, Shimamura K, Rubenstein JL (1998). FGF and Shh signals control dopaminergic and serotonergic cell fate in the anterior neural plate. Cell.

[72] Yan Y, Yang D, Zarnowska ED (2005). Directed differentiation of dopaminergic neuronal subtypes from human embryonic stem cells. Stem Cells.

[73] Joyner AL, Liu A, Millet S (2000). Otx2, Gbx2 and Fgf8 interact to position and maintain a mid-hindbrain organizer. Curr Opin Cell Biol.

[74] Liu A, Joyner AL (2001). Early anterior/posterior patterning of the midbrain and cerebellum. Annu Rev Neurosci.

[75] Schulz TC, Noggle SA, Palmarini GM (2004). Differentiation of human embryonic stem cells to dopaminergic neurons in serum-free suspension culture. Stem Cells.

[76] Schulz TC, Palmarini GM, Noggle SA (2003). Directed neuronal differentiation of human embryonic stem cells. BMC Neurosci.

[77] Roy NS, Cleren C, Singh SK (2006). Functional engraftment of human ES cell-derived dopaminergic neurons enriched by coculture with telomerase-immortalized midbrain astrocytes. Nat Med.

[78] Sonntag KC, Pruszak J, Yoshizaki T (2007). Enhanced yield of neuroepithelial precursors and midbrain-like dopaminergic neurons from human embryonic stem cells using the bone morphogenic protein antagonist noggin. Stem Cells.

[79] Cho MS, Lee YE, Kim JY (2008). Highly efficient and large-scale generation of functional dopamine neurons from human embryonic stem cells. Proc Natl Acad Sci U S A.

[80] Brederlau A, Correia AS, Anisimov SV (2006). Transplantation of human embryonic stem cell-derived cells to a rat model of Parkinson's disease: Effect of in vitro differentiation on graft survival and teratoma formation. Stem Cells.

[81] Ridet JL, Malhotra SK, Privat A (1997). Reactive astrocytes: Cellular and molecular cues to biological function. Trends Neurosci.

[82] Song H, Stevens CF, Gage FH (2002). Astroglia induce neurogenesis from adult neural stem cells. Nature.

[83] Lee DS, Yu K, Rho JY (2006). Cyclopamine treatment of human embryonic stem cells followed by culture in human astrocyte medium promotes differentiation into nestin- and GFAP-expressing astrocytic lineage. Life Sci.

[84] Slaugenhaupt SA, Gusella JF (2002). Familial dysautonomia. Curr Opin Genet Dev.

[85] Troy CM, Brown K, Greene LA (1990). Ontogeny of the neuronal intermediate filament protein, peripherin, in the mouse embryo. Neuroscience.

[86] Fedtsova NG, Turner EE (1995). Brn-3.0 expression identifies early post-mitotic CNS neurons and sensory neural precursors. Mech Dev.

[87] Katz DM (1991). A catecholaminergic sensory neuron phenotype in cranial derivatives of the neural crest: Regulation by cell aggregation and nerve growth factor. J Neurosci.

[88] Brokhman I, Gamarnik-Ziegler L, Pomp O (2008). Peripheral sensory neurons differentiate from neural precursors derived from human embryonic stem cells. Differentiation.

[89] Elkabetz Y, Panagiotakos G, Al Shamy G (2008). Human ES cell-derived neural rosettes reveal a functionally distinct early neural stem cell stage. Genes Dev.

[90] Lamba DA, Karl MO, Ware CB (2006). Efficient generation of retinal progenitor cells from human embryonic stem cells. Proc Natl Acad Sci U S A.

[91] Bhatti MT (2006). Retinitis pigmentosa, pigmentary retinopathies, and neurologic diseases. Curr Neurol Neurosci Rep.

[92] Caraci F, Busceti C, Biagioni F (2008). The Wnt Antagonist, Dickkopf-1, as a target for the treatment of neurodegenerative disorders. Neurochem Res.

[93] Osakada F, Ikeda H, Mandai M (2008). Toward the generation of rod and cone photoreceptors from mouse, monkey and human embryonic stem cells. Nat Biotechnol.

[94] Joannides AJ, Fiore-Hériché C, Battersby AA (2007). A scaleable and defined system for generating neural stem cells from human embryonic stem cells. Stem Cells.

[95] Dhara SK, Hasneen K, Machacek DW (2008). Human neural progenitor cells derived from embryonic stem cells in feeder-free cultures. Differentiation.

[96] Nasonkin IO, Koliatsos VE (2006). Nonhuman sialic acid Neu5Gc is very low in human embryonic stem cell-derived neural precursors differentiated with B27/N2 and noggImplications for transplantation. Exp Neurol.

